# Predicting Bleeding in AML-Associated DIC: Limitations of the ISTH Score and a Modified Approach

**DOI:** 10.3390/diagnostics15233053

**Published:** 2025-11-29

**Authors:** Bedrettin Orhan, Fahir Özkalemkaş, Tuba Bayır, Cumali Yalçın, Büşra Güner, Ezel Elgün, Esra Gülderen, Ayşe Bulur, Tuba Ersal, Fazıl Çağrı Hunutlu, Tuba Güllü Koca, Sinem Çubukçu, Şeyma Yavuz, Vildan Özkocaman

**Affiliations:** 1 Division of Hematology, Department of Internal Medicine, Faculty of Medicine, Bursa Uludag University, Bursa 16059, Turkey; fahir@uludag.edu.tr (F.Ö.); doktorcumali@hotmail.com (C.Y.); elgunezel@hotmail.com (E.E.); tubaersal@uludag.edu.tr (T.E.); fazilhunutlu@gmail.com (F.Ç.H.); tubagullukoca@uludag.edu.tr (T.G.K.); sinemcubukcu@uludag.edu.tr (S.Ç.); seymayavuz2011@hotmail.com (Ş.Y.); vildanoz@uludag.edu.tr (V.Ö.); 2 Department of Biometrics, Faculty of Veterinary Medicine, Fırat University, Elazig 23119, Turkey; tuubabayir@gmail.com; 3 Department of General Internal Medicine, Faculty of Medicine, Bursa Uludag University, Bursa 16059, Turkey; busraguner267@gmail.com (B.G.); esragulderen@uludag.edu.tr (E.G.); aysebulur@uludag.edu.tr (A.B.)

**Keywords:** acute myeloid leukemia, bleeding, disseminated intravascular coagulation, genetics

## Abstract

**Background and Objectives**: Bleeding is a major cause of mortality in cases of acute myeloid leukemia (AML)-associated disseminated intravascular coagulation (DIC). The predictive power of the standard International Society on Thrombosis and Hemostasis (ISTH) score for bleeding in patients diagnosed with AML is limited. This study aimed to evaluate the performance of the standard ISTH score and modified versions in predicting bleeding among acute promyelocytic leukemia (APL, M3) and non-APL AML subgroups. **Methods**: This single-center, retrospective study included 190 AML patients (61 APL, 129 non-APL). The predictive power of the original ISTH score and eight different modified scores—incorporating parameters such as lactate dehydrogenase (LDH) and genetic positivity—for DIC-related bleeding was assessed using receiver operating characteristic (ROC) analysis. **Results**: In the APL group, the original ISTH score was statistically significant in predicting DIC-related bleeding (AUC = 0.727), but the modifications did not improve performance. In the non-APL AML group, the original score did not predict bleeding (AUC = 0.632, *p* = 0.079). However, a modified ISTH score excluding D-dimer and including LDH (≥800 mg/dL) and genetic positivity significantly improved prediction (AUC = 0.710, *p* = 0.005). This modification increased specificity from 48.2% to 60.7% and sensitivity from 76.5% to 82.4%. **Conclusions**: A subtype-specific approach is required to predict bleeding risk in AML-associated DIC. Modified ISTH scores remain suboptimal for APL; however, a modified score incorporating LDH and genetic status represents a promising tool to identify non-APL AML patients at risk of bleeding and warrants prospective validation.

## 1. Introduction

Disseminated intravascular coagulation (DIC) is a syndrome characterized by systemic activation of coagulation, resulting in intravascular generation of thrombin and fibrin, thrombosis of small- to medium-sized vessels, and ultimately organ dysfunction and severe bleeding [1]. DIC always occurs as a response to an underlying disease triggered by thromboinflammatory mediators; it may present acutely in association with sepsis or trauma, or more chronically in association with malignancies. The International Society on Thrombosis and Hemostasis (ISTH) and the Perioperative and Critical Care Thrombosis and Hemostasis subcommittees have listed “acute leukemias” among diseases highly likely to be associated with DIC [2,3]. The association of acute promyelocytic leukemia (APL) with DIC is well established. Similarly, non-APL acute myeloid leukemia (AML) may also be accompanied by DIC [4]. A substantial proportion of patients with APL develop DIC, with reported incidence estimates of 70–80%. In contrast, DIC is observed in approximately 10–20% of non-APL AML patients at diagnosis and during treatment [5]. Although modern APL treatment regimens such as all-trans retinoic acid (ATRA) and arsenic trioxide (ATO) offer high probabilities of long-term remission and disease-free survival, the most common cause of death during induction therapy remains bleeding due to DIC [5]. Catastrophic hemorrhage is the most feared complication during induction, made even more threatening by clinicians’ markedly limited ability to predict and prevent it.

The ISTH DIC scoring system has been widely accepted as a reliable screening tool to detect DIC. Four parameters are evaluated at diagnosis: platelet count, fibrinogen, D-dimer, and prothrombin time [6]. However, while the ISTH score may predict thrombotic complications related to DIC, its ability to predict bleeding is weak [7,8]. Given that bleeding is more common than thrombosis in APL-associated DIC and that early hemorrhagic death remains a major problem among APL and non-APL AML patients presenting with DIC, the ability to predict bleeding is of great importance [2]. A review of studies from 1989 to 2019 identified potential predictors of bleeding in APL patients—including white blood cell count, blast percentage, lactate dehydrogenase (LDH), platelet count, fibrinogen, D-dimer, prothrombin time (PT)/international normalized ratio (INR), activated partial thromboplastin time (aPTT), creatinine, performance status, and differentiation syndrome [2]. However, some studies did not report sensitivity and specificity, and in those that did, ROC analyses yielded low AUC values [8,9,10]. To date, no scoring system with consistently high predictive power for bleeding has been studied.

Therefore, this study aimed to demonstrate whether modifications to the ISTH scoring system could improve the ability to predict DIC-related bleeding in APL and non-APL AML patients.

## 2. Materials and Methods

The records of 483 patients hospitalized with a diagnosis of AML at the Department of Hematology, Bursa Uludag University Hospital between 2000 and 2023 were reviewed retrospectively. According to laboratory tests at diagnosis, 190 patients who had complete data for aPTT, INR, PT, fibrinogen, and D-dimer were included in the study. Bleeding events in patients who presented with DIC were investigated. Patient data included age, sex, AML subtype, administered induction chemotherapy regimens, responses to induction therapies, genetic mutation status at diagnosis, and the parameters that comprise the ISTH score (PT, platelet count, D-dimer, and fibrinogen). Potential parameters considered for the score modifications were recorded as LDH, hemoglobin, and INR. Acute leukemia diagnoses were initially established according to FAB morphology, with subsequent confirmation by immunophenotyping and cytogenetic/molecular studies when available. However, to ensure diagnostic accuracy and specific subgroup identification, final diagnoses were confirmed using immunophenotypic and genetic/molecular testing in accordance with the World Health Organization (WHO) classifications applicable at the time of diagnosis. In this study, APL (AML-M3) was defined morphologically and by the presence of t(15;17) or PML::RARA rearrangement [11]. For non-M3 AML patients, tests for t(8;21), inv(16), t(12;21), FLT3, CEBPA, and NPM1 were evaluated. For APL patients, any genetic positivity other than t(15;17) and for non-M3 AML patients any detected genetic abnormality were classified as genetic+; absence of such abnormalities was classified as genetic−. Response to induction therapy was defined as complete remission according to current AML response criteria, requiring <5% bone marrow blasts, recovery of peripheral blood counts (ANC ≥ 1.0 × 10^9^/L and platelets ≥ 100 × 10^9^/L), and absence of extramedullary disease. Markers of DIC were evaluated before initiation of induction chemotherapy. The diagnosis of DIC was established according to ISTH recommendations [6]. DIC score was calculated as follows: platelet count: >100 × 10^9^/L = 0, <100 × 10^9^/L = 1, <50 × 10^9^/L = 2; D-dimer: no increase (<500 ng/mL) = 0, between 500 and 4000 ng/mL (defined as a moderate increase in the ISTH scoring system) = 2, >4000 ng/mL (defined as a strong increase in the ISTH scoring system) = 3; fibrinogen: >100 mg/dL = 0, <100 mg/dL = 1, and prolonged PT: <3 s = 0, >3 s but <6 s = 1, >6 s = 2. Normal limits were accepted as 11.5–13.5 s for PT, 200–400 mg/dL for fibrinogen, and <500 ng/mL for D-dimer. Patients with an ISTH DIC score ≥ 5 were considered to have overt DIC. DIC-related bleeding events were evaluated as central nervous system hemorrhage, alveolar bleeding, or gastrointestinal bleeding. Major bleeding events were defined as grade 4 bleeding according to the WHO bleeding assessment scale [12].

Eight modifications of the ISTH score were generated; the parameters included in these modifications were platelet count, D-dimer level, fibrinogen, prothrombin time, INR, LDH level (evaluated in two alternative thresholds: >400 or >800 mg/dL), hemoglobin level, and genetic positivity. The parameters incorporated into the eight modified scores were selected based on their identification as independent predictors or significant risk factors in our multivariate logistic regression analysis, as well as their established relevance in AML pathophysiology. Detailed descriptions of the modifications performed are presented in Table 1.

## 3. Results

A total of 190 patients were included in the study. The demographic and clinical characteristics of the patients are presented in Table 2. Of these, 101 patients (53.2%) were female and 89 (46.8%) were male. The median age was 45 years (range, 18–82) for patients with AML-M3 and 49 years (range, 18–83) for those with non-M3 AML. Sixty-one patients (31.2%) were diagnosed with AML-M3, and 129 (67.9%) were diagnosed with non-M3 AML. The FAB distribution of non-M3 AML patients was as follows: 8 patients (4.2%) had M0, 15 (7.9%) had M1, 45 (23.7%) had M2, 28 (14.7%) had M4, 17 (8.9%) had M5, 4 (2.1%) had M6, and 12 (6.3%) had MDS-transformed AML. Induction chemotherapy regimens varied between the groups. In the APL cohort, most patients (91.8%) received the AIDA protocol (ATRA + Idarubicin), while smaller subsets received ATRA + Daunorubicin (6.6%) or ATRA + Cytarabine + Daunorubicin (1.6%). In the non-M3 AML group, the standard ‘3 + 7’ regimen (Idarubicin + Cytarabine) was the predominant therapy (88.4%), followed by Azacitidine-based combinations. Laboratory analysis at diagnosis revealed significantly lower median white blood cell (WBC) counts in APL patients (2690 /mm^3^) compared to non-M3 patients (20,930 /mm^3^) (*p* < 0.001). At diagnosis, DIC with concurrent bleeding was detected in 31 AML-M3 patients (31/61; 50.8%) and in 17 non-M3 AML patients (17/129; 13.2%). Genetic positivity other than t(15;17) was detected in 56 patients (29.5%). Among the patients with genetic positivity, 14 had t(8:21), 7 had t(12:21), 10 had inv16, 16 had FLT3, 15 had NPM1, and 2 had CEBPA mutations. The response rate to induction chemotherapy was 77.9% (148/190). Specific bleeding sites differed between the subgroups. In the APL group, bleeding was primarily gastrointestinal (*n* = 29), but critically, 2 cases of central nervous system (CNS) hemorrhage were observed. In the non-M3 group, bleeding events were predominantly gastrointestinal (*n* = 16), with one case of alveolar hemorrhage. Regarding early outcomes, a total of 17 early deaths occurred during the induction period. Fatal hemorrhage was the primary cause of death in 7 patients (41.2% of all early deaths). Subgroup breakdown showed that fatal bleeding occurred in 5 APL patients (2 CNS, 3 gastrointestinal) and 2 non-M3 patients (both gastrointestinal).

When the predictive power of parameters included and excluded from the ISTH score was evaluated using multivariate regression analysis in patients diagnosed with AML-M3 and DIC (Table 3), prothrombin time, D-dimer, platelet count, LDH, hemoglobin, and INR were not significant predictors (*p* > 0.05). Fibrinogen was significant (*p* = 0.02). ROC analysis of fibrinogen identified a cutoff value of 260 mg/dL. According to the model, the highest sensitivity was observed for fibrinogen (0.935), and the highest specificity was observed for hemoglobin (0.800). When the predictive power of the parameters included and not included in the ISTH score were evaluated with multivariate regression analysis in patients diagnosed with non-M3 AML and DIC (Table 3), prothrombin time, D-dimer, platelet, fibrinogen, hemoglobin, and INR were not significant (*p* > 0.05). LDH was statistically significant (*p* = 0.002). ROC analysis of LDH identified a cutoff value of 897 U/L. In this model, the highest sensitivity was observed for platelet count (0.941), while the highest specificity was observed for LDH (0.845) (Table 3).

The sensitivity and specificity values of the original ISTH score for predicting DIC-related bleeding in AML-M3 and non-M3 AML patients are presented in Table 4. Based on the standard ISTH scoring system (score ≥ 5), overt DIC was identified in 78.7% (48/61) of APL patients and 55.0% (71/129) of non-M3 AML patients at diagnosis, although clinical bleeding events were observed less frequently (50.8% and 13.2%, respectively). Among AML-M3 patients, 29 of 31 patients with bleeding had a high ISTH score, while 11 of 30 patients without bleeding had a low-risk ISTH score. The sensitivity and specificity of the original ISTH score for AML-M3 patients was 93.5% and 36.7%, respectively. Among non-M3 AML patients, 13 of 17 patients with bleeding had high ISTH scores, while 54 of 112 patients without bleeding had low-risk ISTH scores. For this patient group, the sensitivity and specificity of the original ISTH score were calculated as 76.5% and 48.2%, respectively. Modified ISTH Score 3 had a sensitivity of 67% as well as specificity of 66.7% for AML M3 patients and a sensitivity of 70.6% as well as specificity of 66.1% for non-M3 AML patients. With the modified ISTH score 7, sensitivity remained at 67.7% and specificity at 56.7% for AML M3 patients, while sensitivity increased to 82.4% (76.5% in the original score) and specificity increased to 60.7% (48.2% in the original score) for non-M3 AML patients.

**Figure 1:**
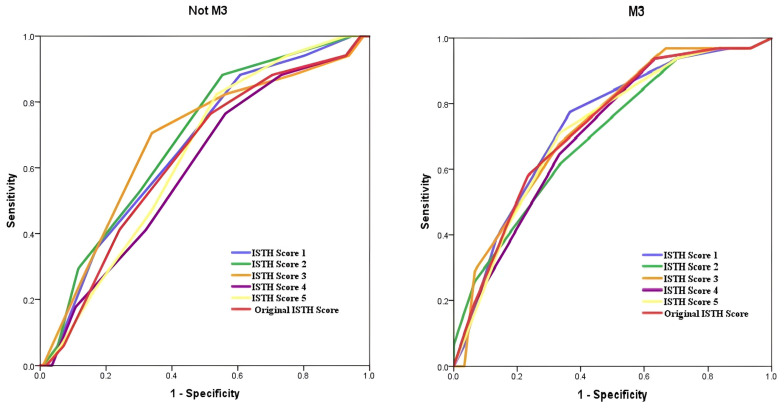
Receiver operating characteristic curves assessing the diagnostic efficacy of models predicting differences between the M3 and non-M3 groups. Combinations with various variants: ISTH Score 1—AUC = 0.665, *p* = 0.029 (non M3), AUC = 0.733, *p* = 0.002 (M3). ISTH Score 2—AUC = 0.689, *p* = 0.012, (non M3), AUC = 0.700, *p* = 0.007 (M3). ISTH Score 3—AUC = 0.680, *p* = 0.017, (non M3), AUC = 0.732, *p* = 0.002 (M3). ISTH Score 4—AUC = 0.600, *p* = 0.185, (non M3), AUC = 0.708, *p* = 0.005 (M3). ISTH Score 5—AUC = 0.638, *p* = 0.068, (non M3), AUC = 0.719, *p* = 0.003 (M3). Original ISTH Score—AUC = 0.632, *p* = 0.079, (non M3); AUC = 0.727, *p* = 0.002 (M3).

**Figure 2:**
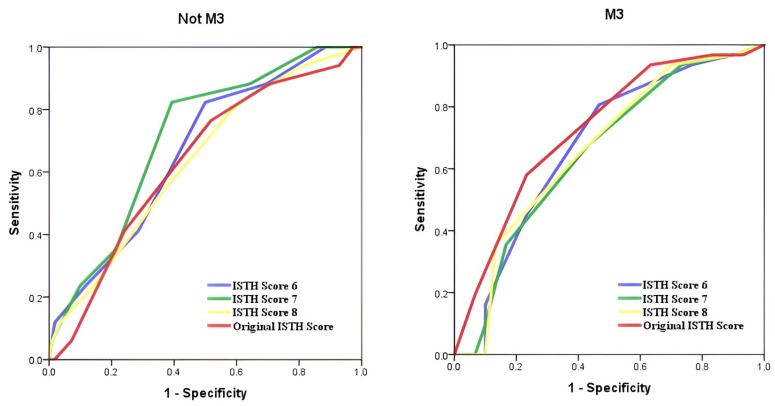
Receiver operating characteristic curves assessing the diagnostic efficacy of models predicting differences between the M3 and non-M3 groups. Combinations with various variants: ISTH Score 6 AUC = 0.666, *p* = 0.028 (non M3), AUC = 0.674, *p* = 0.019 (M3). ISTH Score 7 AUC = 0.710, *p* = 0.005, (non M3), AUC = 0.653, *p* = 0.040 (M3). ISTH Score 8 AUC = 0.636, *p* = 0.072, (non M3), AUC = 0.665, *p* = 0.027 (M3). Original ISTH Score AUC = 0.632, *p* = 0.079, (non M3), AUC = 0.727, *p* = 0.002 (M3).

**Figure 3:**
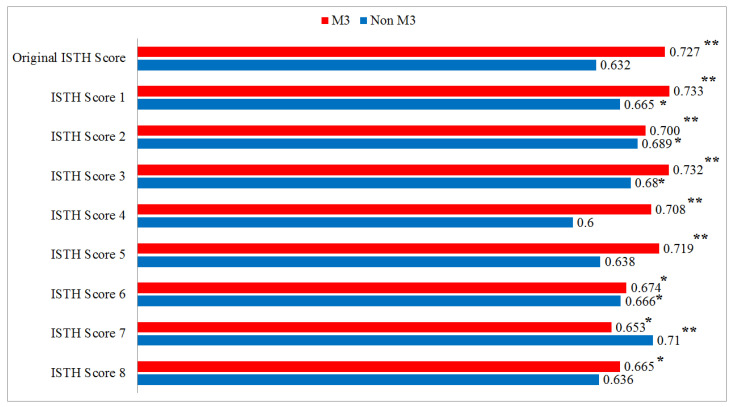
Comparison of AUC values (* *p* < 0.05 and ** *p* < 0.01).

ROC analyses demonstrated that the predictive performance of the original ISTH score for bleeding in AML M3 patients was statistically significant with an AUC of 0.727 (*p* = 0.002), whereas in non-M3 AML patients it was not significant (AUC = 0.632, *p* = 0.079). In AML M3 patients, ISTH modifications 1–5 (including hemoglobin, genetic positivity and LDH) remained statistically significant. In non-M3 AML patients, modifications 1–3 showed modest increases in AUC values (0.665, 0.689, 0.680, respectively) and reached statistical significance (*p* < 0.05). However, ISTH modifications 4 and 5 yielded AUC values of 0.600 and 0.638, respectively, for non-M3 AML patients, and did not reach statistical significance (Figure 1). For ISTH modifications 6–8, statistical significance persisted for AML M3 patients, while ISTH modification 7 (PT, platelet, fibrinogen, LDH, and genetic positivity) had the highest AUC value for non-M3 AML patients and was found to be statistically significant (AUC = 0.710, *p* = 0.005) (Figure 2). A comparison of the original ISTH score and all modifications is presented in Figure 3.

## 4. Discussion

Currently, no laboratory test can be considered a gold standard for the diagnosis of DIC. Therefore, whether in APL or non-APL AML, developing a test or scoring system capable of predicting DIC-related bleeding is inherently challenging. In this study, we investigated the predictive ability for bleeding by modifying the ISTH scoring system. The most notable finding was that in APL patients presenting with DIC, none of the eight modifications enhanced the predictive power of the ISTH score. In non-APL AML patients, the original ISTH score failed to predict bleeding, whereas a modified score incorporating PT, platelet count, fibrinogen, LDH, and genetic positivity significantly improved predictive performance, in terms of AUC and statistical significance (AUC = 0.632, *p* = 0.079 vs. AUC = 0.710, *p* = 0.005).

The proportion of APL patients (32.1%) and non-APL patients (67.9%) in our study provided a more balanced distribution compared to previous reports [8,10]. Consistent with the literature, the present study confirmed that at diagnosis, DIC and bleeding were significantly more frequent in APL patients (50.8%) compared to non-APL AML patients (13.2%). This finding is attributable to the unique pathophysiology of APL and the pronounced coagulopathy induced by the PML::RARα fusion gene [13]. Reported rates of DIC and bleeding in non-APL AML patients range from 10% to 20% in the literature, consistent with the 13.2% rate observed in our cohort [1,5]. For APL patients, DIC-related bleeding rates vary considerably across studies, with some reporting 70–80% and one study reporting as high as 89% [5,14], while another study reporting only 35% [15]. In our study, DIC-related bleeding rate in APL patients was 50.8%. These discrepancies may be explained by differences in study designs and definitions of major bleeding.

Studies evaluating ISTH score thresholds for predicting DIC-related bleeding in APL and non-APL AML patients have produced variable results. A review concluded that although the ISTH score has strong predictive power for thrombosis related to DIC, its ability to predict bleeding is weak [7]. In a study involving non-APL patients, an ISTH score > 4 was associated with a 14-fold increased risk of DIC-related bleeding [16]. In another study involving APL patients, an ISTH score ≥ 6 was reported to predict death due to bleeding [17]. In terms of individual parameters, a study in high-risk APL patients identified elevated WBC count as an independent risk factor for early hemorrhagic death (HR 5.49, *p* < 0.001) [18], and another APL study reported threshold values associated with fatal intracranial bleeding for LDH, INR, and fibrinogen as 423 U/L, 1.37, <121 mg/dL, respectively [19]. In this study, important differences and limitations were observed when evaluating the performance of the standard ISTH scoring system for predicting bleeding in the two AML subgroups. For APL patients, although the standard ISTH score was statistically significant with an AUC of 0.727, its specificity was clinically unacceptably low (36.7%). The test was negative in only one-third of patients without bleeding. This indicates that while the test correctly identified the underlying coagulopathy (biochemical DIC), these patients did not manifest with major clinical hemorrhage. Therefore, regarding the specific endpoint of ‘major bleeding prediction,’ the specificity was limited. In non-APL AML patients, while the sensitivity of the standard ISTH score was higher (76.5%), its specificity was quite low (48.2%), and the AUC value (0.632) did not reach statistical significance (*p* = 0.079). These findings strongly support that the standard ISTH score, originally developed for conditions like sepsis [6,20,21], remains suboptimal for predicting bleeding risk in AML subtypes and requires modifications.

The coagulopathy specific to APL is known to have a unique pathophysiology, triggered by massive tumor cell lysis, overexpression of procoagulants like annexin II and tissue factor, and characterized by intense hyperfibrinolysis [22]. This may create a “ceiling effect” where standard DIC parameters (particularly D-dimer) are already markedly elevated at diagnosis. Consequently, the parameters added to the existing scoring system likely failed to provide additional discriminatory information within this dominant and complex coagulopathy mechanism. Indeed, in our study, none of the eight different modifications made for APL patients significantly enhanced the predictive power of the original ISTH score. The most notable finding of the study was that in non-APL AML patients, the modified ISTH Score 7, in which D-dimer was excluded and replaced with LDH (threshold of ≥800 mg/dL) and genetic positivity, significantly enhanced predictive ability for bleeding (AUC = 0.710, *p* = 0.005). This modification not only improved the AUC but also yielded a more balanced predictive model, increasing sensitivity from 76.5% to 82.4% and specificity from 48.2% to 60.7%.

The success of this modification can be explained by several key mechanisms. First, lactate dehydrogenase (LDH) is a well-established marker of high tumor burden and aggressive cell kinetics in AML [23]. Elevated LDH levels reflect intense leukocytolysis, leading to the release of procoagulant microparticles and cytokines, which in turn trigger endothelial damage and coagulation activation [24]. Indeed, in our multivariable analysis, LDH was the only significant predictor of bleeding in non-APL AML (*p* = 0.002).

The fact that 5 of 7 fatal hemorrhages occurred in APL patients in our series mirrors large APL cohorts in which hemorrhagic early death remains the dominant cause of induction failure, despite modern ATRA-based regimens [18]. By contrast, fatal bleeding was less frequent but still clinically relevant in non-APL AML, emphasizing that DIC-related coagulopathy is not restricted to APL and may also contribute to early mortality in other AML subtypes. Together with previous reports, our findings support the concept that immediate recognition of high-risk patients and protocolized hemostatic management are crucial to reduce early hemorrhagic death in acute leukemia [5].

Second, the inclusion of the “genetic positivity” parameter is important because it incorporates disease biology and heterogeneity into the scoring system. In our cohort, the rate of genetic positivity (excluding t(15;17)) was 29.5% (56/190), with a significant distribution difference between M3 and non-M3 groups (9 vs. 47; *p* = 0.002). Subgroup analysis revealed significant distribution differences for t(8;21) (*p* = 0.04) and inv(16) variants (*p* = 0.03), but not for t(12;21) (*p* = 1.00). These findings suggest that genetic-based coagulopathy patterns may be heterogeneous, consistent with literature reports describing different coagulation/fibrinolysis profiles across AML subtypes. Certain genetic mutations, such as FLT3-ITD, have been shown to be associated with higher leukocyte counts and aggressive disease course, thus may increase susceptibility to coagulopathy [4,25]. FLT3-ITD mutation is associated with a high blast burden, increased proliferation rate, and procoagulant microparticles released into the circulation due to apoptosis [16]. These particles can trigger thrombin generation by increasing tissue factor expression, which may elevate the risk of microthrombosis and consumption coagulopathy-related bleeding. NPM1 mutation is characterized by altered nucleolar-protein localization and cell cycle irregularities [4]. Including this parameter may have enhanced the predictive power of the score by acting as a surrogate marker reflecting the aggressiveness of the disease at the molecular level. Although NPM1 mutations are typically associated with favorable long-term prognosis, in the context of acute coagulopathy, they are often associated with high tumor burden, rapid cell turnover, and hyperleukocytosis (particularly when co-mutated with FLT3), thereby contributing to a pro-coagulant state [26]. Finally, the exclusion of D-dimer from the model is a noteworthy finding. While D-dimer is a highly sensitive marker of coagulation activation, it is influenced by multiple factors in AML, including inflammation and cell destruction, making it a less specific predictor of bleeding [24]. Moreover, it may be more closely associated with thrombosis risk and may not specifically reflect bleeding risk. Therefore, replacing D-dimer with LDH and genetic positivity—biological markers more directly tied to AML pathophysiology—appears to have enhanced the bleeding-specific predictive accuracy of the score in non-APL AML.

## 5. Conclusions

This study demonstrates that a “one-score-fits-all” approach is inadequate for predicting bleeding risk in AML-associated DIC. The standard ISTH score performed suboptimally in predicting bleeding risk in APL patients and the modifications showed no significant improvement, a modified score incorporating LDH and genetic positivity while excluding D-dimer (Modified ISTH Score 7) provided a significant and clinically meaningful improvement in predictive accuracy for non-APL AML patients. These findings highlight the need for AML subtype-specific, pathophysiology-driven risk scoring systems. In this study, all mutations were analyzed collectively, but the individual predictive contributions of different genetic subtypes should be explored in larger cohorts. Modified ISTH Score 7 may represent a simple and effective tool for identifying non-APL AML patients at high risk of bleeding at diagnosis. However, these results highlight the need for prospective validation and may guide the development of AML subtype-specific bleeding risk models.

### Limitations

This study has several important limitations that warrant consideration. First, the retrospective design of the study led to incomplete data capture; only 190 of the initial 483 patients with full datasets could be analyzed. This introduces a potential risk of selection bias. Second, the “genetic positivity” parameter, which contributed to the success of the model in non-APL AML, grouped together biologically distinct mutations such as t(8;21) and FLT3. This simplification may have masked the unique contributions of individual genetic subtypes to bleeding risk. Finally, important confounding factors such as infections, which may trigger or exacerbate DIC, were not excluded in our analyses, warranting cautious interpretation of the results.

## Figures and Tables

**Table 1:** Modifications of ISTH score and parameters.

**Parameters**									
**Modifications**	**Prothrombin Time**	**D-Dimer**	**Fibrinogen**	**Platelet**	**LDH**	**INR**	**Hgb**	**Genetics**	**Total Score**
**ISTH Score 1**	<3 = 0 >3 but <6 = 1 >6 = 2	<500 ng/mL = 0 500–4000 ng/mL = 2 >4000 ng/mL = 3	>100 mg/dL =0, <100 mg/dL = 1	>100 × 10^9^/L = 0 <100 × 10^9^/L = 1 <50 × 10^9^/L = 2	<400 mg/dL = 0 ≥400 mg/dL = 1				**≥6**
**ISTH Score 2**	<3 = 0 >3 but <6 = 1 >6 = 2	<500 ng/mL = 0 500–4000 ng/mL = 2 >4000 ng/mL = 3	>100 mg/dL =0, <100 mg/dL = 1	>100 × 10^9^/L = 0 <100 × 10^9^/L = 1 <50 × 10^9^/L = 2	<800 mg/dL = 0 ≥800 mg/dL = 1				**≥6**
**ISTH Score 3**	<3 = 0 >3 but <6 = 1 >6 = 2	<500 ng/mL = 0 500–4000 ng/mL = 2 >4000 ng/mL = 3	>100 mg/dL =0, <100 mg/dL = 1	>100 × 10^9^/L = 0 <100 × 10^9^/L = 1 <50 × 10^9^/L = 2				Any positivity = 1	**≥6**
**ISTH Score 4**	<3 = 0 >3 but <6 = 1 >6 = 2	<500 ng/mL = 0 500–4000 ng/mL = 2 >4000 ng/mL = 3	>100 mg/dL =0, <100 mg/dL = 1	>100 × 10^9^/L = 0 <100 × 10^9^/L = 1 <50 × 10^9^/L = 2			≥7 = 0 <7 = 1		**≥6**
**ISTH Score 5**	<3 = 0 >3 but <6 = 1 >6 = 2	<500 ng/mL = 0 500–4000 ng/mL = 2 >4000 ng/mL = 3	>100 mg/dL =0, <100 mg/dL = 1	>100 × 10^9^/L = 0 <100 × 10^9^/L = 1 <50 × 10^9^/L = 2		≤1.2 = 0 >1.2 = 1			**≥6**
**ISTH Score 6**	<3 = 0 >3 but <6 = 1 >6 = 2		>100 mg/dL =0, <100 mg/dL = 1	>100 × 10^9^/L = 0 <100 × 10^9^/L = 1 <50 × 10^9^/L = 2	<400 mg/dL = 0 ≥400 mg/dL = 1			Any positivity = 1	**≥5**
**ISTH Score 7**	<3 = 0 >3 but <6 = 1 >6 = 2		>100 mg/dL =0, <100 mg/dL = 1	>100 × 10^9^/L = 0 <100 × 10^9^/L = 1 <50 × 10^9^/L = 2	<800 mg/dL = 0 ≥800 mg/dL = 1			Any positivity = 1	**≥5**
**ISTH Score 8**	<3 = 0 >3 but <6 = 1 >6 = 2		>100 mg/dL =0, <100 mg/dL = 1	>100 × 10^9^/L = 0 <100 × 10^9^/L = 1 <50 × 10^9^/L = 2				Any positivity = 1	**≥4**
**Original Score**	<3 = 0 >3 but <6 = 1 >6 = 2	<500 ng/mL = 0 500–4000 ng/mL = 2 >4000 ng/mL = 3	>100 mg/dL =0, <100 mg/dL = 1	>100 × 10^9^/L = 0 <100 × 10^9^/L = 1 <50 × 10^9^/L = 2					**≥5 overt DIC**

ISTH: International Society on Thrombosis and Hemostasis; LDH: lactate dehydrogenase; INR: international normalized ratio; Hgb: hemoglobin; ng: nanogram; mg: milligram; mL: milliliter, dL: deciliter; L: liter; DIC: disseminated intravascular coagulation.

**Table 2:** Demographic and clinical characteristics of the patients.

	AML-M3	Non-M3
**Sex**		
*Female*	33 (54.1%)	68 (52.7%)
*Male*	28 (45.9%)	61 (47.3%)
**Age**	45 (18–82)	49 (18–83)
**AML Subgroups**	M3 61 (32.1%)	M0: 8 (4.2%)
		M1: 15 (7.9%)
		M2: 45 (23.7%)
		M4: 28 (14.7%)
		M5: 17 (8.9%)
		M6: 4 (2.1%)
		MDS trans. AML: 12 (6.3%)
**Chemotherapy**		
*AIDA*	56 (91.8%)	-
*ATRA + Daunorubicin*	4 (6.6%)	-
*ATRA + Cytarabine + Daunorubicine*	1 (1.6%)	-
*3 + 7*	-	114 (88.4%)
*Azacitidine + Venetoclax*	-	7 (5.4%)
*Azacitidine*	-	4 (3.1%)
*2 + 5*	-	3 (2.3%)
*ARA-C + Dexamethasone*	-	1 (0.8%)
**Laboratory Findings, Median (Min-Max)**		
*WBC (mm* ^3^ *)*	2690 (610–161,600)	20,930 (600–316,000)
*Hemoglobin (g/dL)*	9.7 (8.4–11.4)	8.7 (7.5–9.8)
*Platelet count (×10* ^9^ */L)*	30.6 (19.3–49.8)	48.2 (29.3–85.5)
*Fibrinogen (mg/dL)*	167 (106–275)	378 (309–483)
*D-dimer (mg/L)*	13.5 (6.0–31.1)	2.37 (1.13–6.68)
**DIC with bleeding**	31/61 (50.8%)	17/129 (13.2%)
**(# of patients)**		
*GIS*	29	16
*CNS*	2	0
*Alveolar*	0	1
**Any genetic positivity** (excluding t(15;17) *)	56/190 (29.5%)	
	9	47 **
t(8;21)	1	13
t(12;21)	2	5
inv16	-	10
FLT3	4	12
NPM1	2	13
CEBPA	0	2
**Response to induction**	148/190 (77.9%)	

AML: acute myeloid leukemia; 2 + 5: idarubicin and cytarabine; 3 + 7: idarubicin and cytarabine; AIDA: All trans retinoic acid, idarubicine, cytarabine; ATRA: All trans retinoic acid; ARA-C: cytarabine and arabinoside; t: translocation; inv: inversion; CNS: central nervous system; FMS-like tyrosine kinase 3; GIS: gastrointestinal system; MDS: myelodysplastic syndrome; NPM1: Nucleophosmin 1; CEBPA: CCAAT enhancer binding protein alpha; DIC: disseminated intravascular coagulation; WBC: white blood cell. * The sum of individual genetic subgroups may exceed the total number of genetically positive patients due to the presence of co-mutations in single individuals. Specifically, in the Non-M3 group, co-occurrences included t(8;21) with t(12;21) (*n* = 5), FLT3 with NPM1 (*n* = 2), and NPM1 with CEBPA (*n* = 1). In the APL group, secondary mutations accompanying t(15;17) included FLT3 (*n* = 4), t(12;21) (*n* = 2), and NPM1 (*n* = 2). ** *p* = 0.002.

**Table 3:** Multivariate logistic regression model of predictors.

	Parameter	Median (IQR)	AUC (95% CI)	Cutoff	Sensitivity	Specificity	*p*-Value
**M3**	PT	13.80 (12.60–15.15)	0.639 (0.499–0.779)	13.45	0.742	0.567	0.149
	D-Dimer (mg/L)	13.50 (5.99–31.13)	0.510 (0.363–0.658)	14.60	0.419	0.467	0.785
	PLT (10^3^)	30.6 (19.3–49.8)	0.564 (0.415–0.713)	52.95	0.871	0.333	0.134
	Fibrinogen (mg/dL)	167.00 (106.00–274.50)	0.727 (0.600–0.854)	260.00	0.935	0.467	0.02
	LDH (U/L)	321.00 (219.75–536.50)	0.594 (0.450–0.739)	313.00	0.667	0.600	0.244
	HGB (g/dL)	9.70 (8.40–11.40)	0.531 (0.384–0.679)	8.75	0.355	0.800	0.737
	INR	1.13 (1.04–1.35)	0.628 (0.487–0.770)	1.08	0.871	0.433	0.118
**Non-M3**	PT	12.90 (12.05–14.00)	0.553 (0.401–0.705)	13.55	0.529	0.643	0.552
	D-Dimer (mg/L)	2.37 (1.13–6.68)	0.572 (0.428–0.716)	4.85	0.471	0.688	0.822
	PLT (10^3^)	48.2 (29.3–85.5)	0.621 (0.484–0.758)	79.35	0.941	0.330	0.073
	Fibrinogen (mg/dL)	378.00 (308.50–482.50)	0.565 (0.413–0.717)	351.50	0.652	0.588	0.491
	LDH (U/L)	478.00 (257.00–868.00)	0.756 (0.634–0.877)	897	0.647	0.845	0.002
	HGB (g/dL)	8.70 (7.47–9.75)	0.517 (0.389–0.644)	7.47	0.118	0.732	0.758
	INR	1.10 (1.00–1.20)	0.559 (0.426–0.692)	1.18	0.471	0.678	0.653

CI, confidence interval; IQR, interquartile range; AUC, area under the curve; LDH, lactate dehydrogenase; INR, international normalized ratio; HGB, hemoglobin; mg, milligram; mL, milliliter; dL, deciliter; L, liter; PT, prothrombin time; PLT, platelet; U, unit; g, gram.

**Table 4:** Sensitivity and specificity of the ISTH score.

			Real Test (Bleeding)			Sensitivity (%)	Specificity (%)
			Positive	Negative	Total		
**Original ISTH score**	Non-M3	Positive	13	58	71	76.5	48.2
		Negative	4	54	58		
		Total	17	112	129		
	M3	Positive	29	19	48	93.5	36.7
		Negative	2	11	13		
		Total	31	30	61		
**ISTH Score 3**	Non-M3	Positive	12	38	50	70.6	66.1
		Negative	5	74	79		
		Total	17	112	129		
	M3	Positive	21	10	31	67.7	66.7
		Negative	10	20	30		
		Total	31	30	61		
**ISTH Score 7**	Non-M3	Positive	14	44	58	82.4	60.7
		Negative	3	68	71		
		Total	17	112	129		
	M3	Positive	21	13	34	67.7	56.7
		Negative	10	17	27		
		Total	31	30	61		

ISTH: International Society on Thrombosis and Hemostasis.

## Data Availability

The data that support the findings of this study are available on request from the corresponding author, B.O. The data are not publicly available due to ethical reasons.
